# Supplementary material for: Dog Population & Dog Sheltering Trends in the United States of America
Source: Animals (Basel). 2018 Apr 28;8(5):68. doi: 10.3390/ani8050068 (PMC5981279; doi:10.3390/ani8050068)
Supplement: Supplementary file 1 [file animals-08-00068-s001.docx]

# Supplementary Material

S1. Data retrieval and analysis

Table 1: Source of data for Review

| Data Source | Comments |
| --- | --- |
| Individual Shelters (e.g. LA Dept of Animal Regulation & San Mateo County [1]) | Data collection and reporting will vary with different management teams and individual shelters do not include all shelters/rescues in a community so there will be gaps but these and the one below for NY City (compiled from ASPCA and ACC of NY reports [2,3]) are the only data sets that go back into the 1970s. |
| Clifton analyses in *Animal People* [4] | Merritt Clifton has been reporting on shelter intake numbers and shelter euthanasia using a standardized “animals per 1,000 people” metric for more than twenty-five years. He has also unearthed earlier surveys of dog and cat demographics to provide some context. His data agree with the trends reported in this review. |
| State data sets (e.g. CA, NJ, NH) [5, 6, 7] | Several US states have been collecting data on shelter intake and outcomes over a period of time. The California data goes back to 1997 [5] while the New Jersey data stretches further back to 1984 [5]. The raw data suffers from problems (some communities do not report every year or their reports are incomplete) but the data sets can nevertheless be used with appropriate caveats to establish state-wide trends over the period that data has been collected. |
| Marsh online book [7] | Peter Marsh produced an online book (*Getting to Zero*) in 2012 (<http://www.shelteroverpopulation.org/Books/Getting_to_Zero.pdf>). He emphasized the importance of data in designing and implementing shelter programs and provided several data sets including one for New Hampshire. It is assumed that this data set is internally consistent and provides an accurate picture of trends in New Hampshire. |
| Petpoint reports [8] | PetHealth provides shelter management software (Petpoint) at no cost to animal shelters and animal rescue operations in North America. The data generated by shelter record-keeping of intake and outcomes is stored on servers maintained by PetHealth. They then produce monthly compilations of the data from around 50% of the 2,000+ shelters and rescues who are currently using their software. [It is not clear why they do not provide data from all of the entities using their software.] Nevertheless, it is possible to use these monthly data reports to track intake and outcome trends from 2009 to the present. We have standardized these monthly totals by calculating the numbers for 1,000 entities for each month so that we can compare a standardized number by month and year for a representative sample of 1,000 Petpoint users. However, the Petpoint users are NOT a representative sample of US shelters and rescues. Municipal animal control shelters are reticent about using the software because data on their constituents are then stored on the servers of a commercial vendor. However, some municipalities do use Petpoint. We believe the sample size is large enough to provide representative trend data. It appears that the calculated annual intakes and outcomes for 1,000 Petpoint users represent around 20% of the total shelter and rescue intake in the United States. |
| HSUS Surveys (unpublished) | The Humane Society of the United States surveyed shelter animal intake and animal euthanasia in 1973 and 1981 and extrapolated to provide national estimates. The surveys involved around 300 shelters and their catchment areas (human populations) and then these numbers were extrapolated to provide national estimates. |
| Other data sources | There are episodic analyses of shelter intake and outcomes by states and individual entities across the US. For example, Linda Lord (Ohio State University) [9] published papers and reports of two surveys of Ohio shelters in 1996 and 2004. In 2016, researchers from Mississippi State produced a report of dog intake and outcome into shelters across the United States [10]. Their data is in reasonably good agreement with the national estimates produced from the Petpoint data (generated by multiplying the 1,000-entity data set by 5). |
| Data sources not used | The American Humane Association (AHA) published estimates of shelter animal intake and outcome from 1985-1988 and 1990. These have not been included in this analysis because of methodological issues. AHA typically sent out a short questionnaire to around 6,000 entities on its mailing list (only about half of which were likely to be shelters). It then received from 100 to 300 (n) responses. They multiplied the total numbers of animal intakes and outcomes by 6,000/n in order to obtain a national estimate of animal intake and euthanasia. There were no corrections to account for size of the entities responding compared to the size of the non-respondents, nor for the proportion of the 6,000 entities who were not actually shelters. The Maddies Fund [11] also has been reporting on the intake/outcome trends for a number of communities across the USA. The trends indicated by these reports match the trends reported in this analysis but we did not make use of these data. The National Council on Pet Population Study and Policy (NCPPSP) was formed in 1993 [12] .They conducted four national surveys of shelter intake and outcome but response rates were low and the results were not considered (by the NCPPSP) to be representative of the US as a whole. |

S2. More on the PetPoint dataset [8]

PetPoint is an industry program provided for free to shelters and rescues participating in the 24PetWatch Insurance and Microchip program. It is cloud-based software and PetPoint publishes monthly and annual reports of the data generated by their program on their website. The number of participating shelters on which data is reported varies over time (from around 900 shelters and rescues initially to over 1,250 in the most recent reports). It is possible using these monthly reports to generate a data set that is “standardized” for 1,000 entities over time (2009 to the present). The sample tends to be light in animal control agencies (municipalities are reluctant to allow municipal data to be stored on private servers as is the case with the PetPoint data) but it nevertheless provides a very useful indication of trends in shelter intake and outcomes for the United States.

Figure 1: Percentage of dogs adopted, RTO and euthanized nationwide based on PetPoint data September 2009-September 2017 [8], and national dog population estimates between 2010 and 2016 all based on the APPA reports accept 2011 is taken from the AVMA report


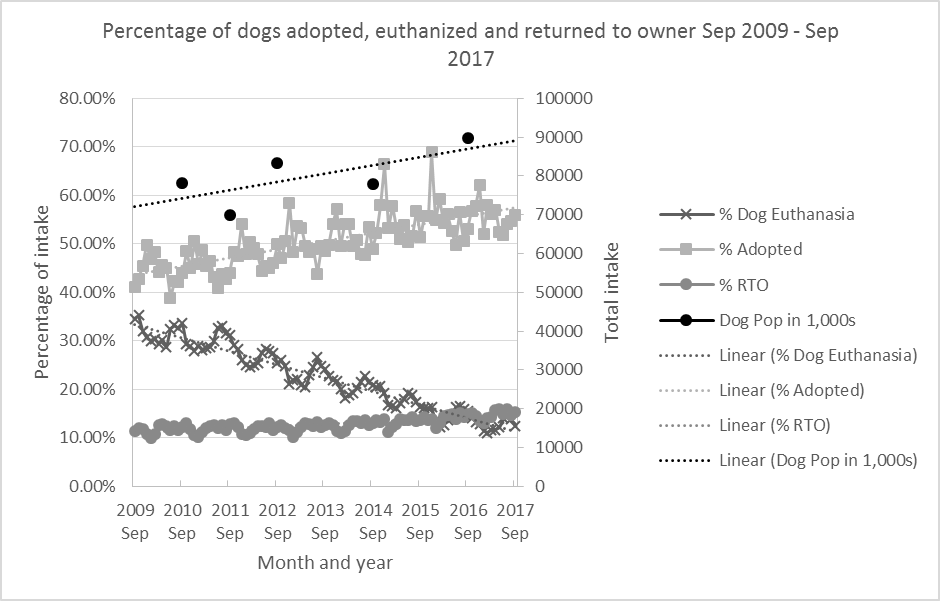


S3. Comparison of different national dog population and shelter number estimates

To estimate the number of shelters in the United States Woodruff and Smith [10] used a modified version of the capture - recapture method used in wild animal surveys. With a simple Lincoln-Petersen method they used two sampling frameworks to sample from the same population. Simply described, a mark-recapture exercise is two counting events of the same population in which M animals are marked in the first count and C animals captured in the second count, which includes R marked animals from the first count. Based on this we can estimate the size of the population by using the formula N=MC/R.

In their case they compared the results of two sampling frames in which they sampled shelters and rescues by calling them. This mark-recapture was conducted in Florida, South Carolina, Maine, Michigan and Mississippi.

| State | W&S Est | All APOs (Guidestar D20) | APOs with $110K+ income (Guidestar) | Estimated Shelter Number (Other sources) | Source of Estimate in Previous Column |
| --- | --- | --- | --- | --- | --- |
| Florida | 190 | 1,684 | 205 | 116 | Levy, personal info |
| South Carolina | 158 | 369 | 57 |  |  |
| Maine | 95 | 151 | 26 | 32 | From state sources, plus 16 counties with pounds |
| Michigan | 214 | 634 | 81 | 151 | Michigan Survey, 83 counties, 65 private orgs with $100K+ |
| Mississippi | 92 | 154 | 19 | 23 | Private orgs with budgets of more than $100K |
| USA | 7,067 | 25,316 | 3,196 | 3,350 – 3,500 | Estimate from in-house HSUS survey in 2004-2005. |

The above table compares the Woodruff and Smith estimates for total US shelters (as extrapolated from their five selected states) with three different numbers. The first column simply identifies the number of animal protection groups (D20 classification by the Internal Revenue Service) in each state taken from the Guidestar data base accessed on November 29, 2017 (Guidestar is a comprehensive database for the 1.6 million NGOs in the US) [13]. The second column identifies the number of Animal NGOs (this does NOT include municipal shelters) in each state who had annual income of $110,000 or more in the most recent reporting year (usually 2015). We assume that any shelter would need at least $110K in annual income to hire at least one staff person and support a structure that took in unwanted animals into a building owned and managed by the organization. Small shelters (those that take in a few hundred animals or less a year) typically contribute less than ten percent (usually around 5%) of overall State shelter intake and euthanasia. As is evident in the table, there are tens of thousands of small animal NGOs in the USA with less than $110,000 in income and a majority have less than $10,000 in annual income.

About 40% of all shelters are municipal operations that would not be found in the Guidestar database. The last column in the table identifies other sources of the total number of shelters. For example, Dr Julie Levy at the University of Florida identified every shelter (housing animals in their own building) in the state of Florida and produced a tally of 116 (personal communication, Julie Levy, 2017) compared to Woodruff and Smith’s estimate of 190 shelters. The table above identifies 205 animal NGOs in Florida with income of $110,000 or more but only about half of these are shelters. Florida has seen an explosion of small rescue groups in the past decade and we suspect most of the “non-shelters” fall into this category. In Maine, there are 16 recognized shelters in the state plus a similar number of city and county pounds that handle small numbers of animals. So there may be as many as 30-35 “shelters” but Woodruff and Smith estimate there are 95. In Michigan, there are 81 animal NGOs with income of $110,000 plus and 81 counties (some/many of which will have shelters). Surveys of Michigan shelters in the past decade indicate there may be as many as 150 shelters in the state [14]. Woodruff and Smith estimate there are 214 shelters in Michigan.

We conclude from the above that the Woodruff and Smith estimate of the total number of shelters in the USA is high and that our estimate of 3,500 is closer to the actual number. If there are more, they are likely to be very small operations that do not contribute significantly to national shelter intake and outcomes.

S4. Variance of total dog populations measured against total human populations in the USA and selected states.

The AVMA (American Veterinary Medical Association) [15] has conducted a national survey of the pet population every five years and since 1986 has been reporting sufficient data to produce state-by-state estimates of dog populations in each state. The APPA (American Pet Products Association) has been conducting biannual surveys of the US pet population since 1988 [16]. Although both organizations used similar methodology over much of this period, their national estimates of total dog population differed, sometimes by a significant amount. In general, the APPA estimates of national dog population were higher than the AVMA’s (figures 2 & 3).

Figure 2. AVMA and APPA estimates of national pet dog populations over time [15, 16]

Figure 3. Relative number of national pet dogs in the US over time (data from AVMA and APPA surveys) [15, 16].

The AVMA data also provides opportunities to tally the number of pet dogs in specific states and indicates significant differences in dog ownership rates from one state to another. These differences are likely to be relevant in assessing shelter intake and outcome but there are no published reports that even reference the state by state differences in pet dog numbers.

In the tables below, we selected Southern and Northeastern States and their respective dogs per 100 people ratios. There are significant variations (see tables 3-5). For example, Massachusetts has 12 dogs per 100 people while New Mexico has 34 dogs per 100 people. The national average of dogs per 100 people is 23 [15]. It is unclear what drives these differences. There is limited correlation between dog ownership rates and shelter euthanasia rates.

Table 3: Dog ownership rate in dogs/ 1,000 people for New England states (compiled from [15]).

| State | 1996 | 2001 | 2006 | 2011 | Average |
| --- | --- | --- | --- | --- | --- |
| Connecticut | 152 | 151 | 155 | 142 | 150 |
| Maine | 158 | 242 | 209 | 226 | 209 |
| Massachusetts | 124 | 106 | 136 | 129 | 124 |
| New Hampshire | 128 | 156 | 200 | 161 | 161 |
| Rhode Island | 129 | 134 | 200 | 153 | 154 |
| Vermont | 186 | 207 | 2.4 | 227 | 223 |

Table 4: Dog ownership rate in dogs/ 1,000 people for South Atlantic states (compiled from [15]).

| State | 1996 | 2001 | 2006 | 2011 | Average |
| --- | --- | --- | --- | --- | --- |
| Delaware | 184 | 227 | 168 | 180 | 190 |
| Florida | 191 | 202 | 2.5 | 221 | 215 |
| Georgia | 229 | 239 | 275 | 253 | 249 |
| Maryland | 150 | 181 | 178 | 157 | 167 |
| North Carolina | 255 | 274 | 304 | 261 | 274 |
| South Carolina | 248 | 264 | 290 | 255 | 264 |
| Virginia | 202 | 216 | 214 | 210 | 211 |
| Washington DC | 50 | 39 | 69 | 66 | 56 |
| West Virginia | 294 | 411 | 356 | 338 | 350 |

Table 5: Dog ownership rate in dogs/ 1,000 people for Mountain states (compiled from [15]).

| State | 1996 | 2001 | 2006 | 2011 | Average |
| --- | --- | --- | --- | --- | --- |
| Arizona | 216 | 244 | 300 | 277 | 259 |
| Colorado | 245 | 262 | 297 | 264 | 267 |
| Idaho | 247 | 291 | 327 | 225 | 272 |
| Montana | 258 | 313 | 372 | 283 | 306 |
| Nevada | 224 | 242 | 270 | 212 | 237 |
| New Mexico | 292 | 289 | 449 | 338 | 342 |
| Utah | 181 | 159 | 175 | 146 | 165 |
| Wyoming | 285 | 376 | 447 | 220 | 332 |

If we compare dogs per 100 people as a function of human density, there is an inverse relationship between the owned dog population of a state and the number of people per square kilometer. [We find a similar inverse relationship between relative dog numbers and human density across the globe.]

References

1. Anonymous LA Animal Services Statistical Reports. 2007 – 2017 and from Annual Reports of Local Rabies Control Activities, California Department of Health and Human Services from 1997 to the present – see https://www.cdph.ca.gov/Programs/CID/DCDC/Pages/LocalRabiesControlActivities.aspx. Note, the reports for the years from 1997 to 2009 are no longer available on the website.
2. Zawistowski, S., et al. Population dynamics, overpopulation, and the welfare of companion animals: new insights on old and new data. *Journal of Applied Animal Welfare Science*, **1998**, 1. Jg., Nr. 3, S. 193-206.
3. Animal Care Centers of NYC. Available online: <http://www.nycacc.org/about/statistics> (accessed 03/27/2018).
4. Clifton, M. Record low shelter killing raises both hopes & questions. Available online: <http://www.animals24-7.org/2014/11/14/record-low-shelter-killing-raises-both-hopes-questions/>. (Accessed on 30/10/2017).
5. Anonymous. Annual Reports of Local Rabies Control Activities, California Department of Health and Human Services from 1997 to the present – see https://www.cdph.ca.gov/Programs/CID/DCDC/Pages/LocalRabiesControlActivities.aspx.
6. Stray Animal Intake and Disposition Survey: Dogs and Cats 1984-2016. New Jersey Department of Health: Infectious and Zoonotic Disease Program. <http://www.nj.gov/health/vph/2017_yearly_summary_dogs_cats.pdf> Accessed March 27, 2018.
7. Marsh, P. Replacing myth with math: using evidence-based programs to eradicate shelter overpopulation. Town and Country Reprographics, Incorporated. **2010**.
8. Pet Point. Industry data, retrieved from: http://www.petpoint.com/industry_data.asp. (Accessed on: Sep 2009 - June 2017).
9. Lord, L. K., Wittum, T. E., Ferketich, A. K., Funk, J. A., Rajala-Schultz, P., & Kauffman, R. M. Demographic trends for animal care and control agencies in Ohio from 1996 to 2004. *Journal of the American Veterinary Medical Association*, **2006**, *229.1*, 48-54.
10. Woodruff, K.A. & Smith D.R. An Estimate of the Number of Dogs in US Shelters. Proceedings at NAVC. **2017**.
11. Searchable Database. Available online: <http://www.maddiesfund.org/searchable-database.htm> (accessed on 03/27/2018).
12. National Council on Pet Population. Available online: <http://www.sawanetwork.org/?page=nationalcouncil> (accessed on 03/27/2018)
13. Guidestar database. Available online: <http://www.guidestar.org/Home.aspx> (accessed 11/29/2018)
14. Michigan Department of Agriculture and Rural Development. Animal shelter annual reports. Available online: <http://www.michigan.gov/mdard/0,4610,7-125-1569_16979_21260---,00.html>. (Accessed on 15/10/2016)
15. American Veterinary Medical Association (AVMA). Pet survey (2012 Edition).
16. American Pet Products Association (APPA) Biennial National pet Owners Surveys. See <http://www.americanpetproducts.org/pubs_survey.asp> for more recent survey.
